# Towards Precision Engineering of Canonical Polyketide Synthase Domains: Recent Advances and Future Prospects

**DOI:** 10.3390/molecules22020235

**Published:** 2017-02-05

**Authors:** Carmen L. Bayly, Vikramaditya G. Yadav

**Affiliations:** 1 Department of Genome Sciences & Technology, The University of British Columbia, Vancouver, BC V5Z 4S6, Canada; c.bayly@alumni.ubc.ca; 2 Department of Chemical & Biological Engineering, The University of British Columbia, Vancouver, BC V6T 1Z3, Canada

**Keywords:** polyketide synthases, structural biology, protein engineering, synthetic biology, antibiotics

## Abstract

Modular polyketide synthases (mPKSs) build functionalized polymeric chains, some of which have become blockbuster therapeutics. Organized into repeating clusters (modules) of independently-folding domains, these assembly-line-like megasynthases can be engineered by introducing non-native components. However, poor introduction points and incompatible domain combinations can cause both unintended products and dramatically reduced activity. This limits the engineering and combinatorial potential of mPKSs, precluding access to further potential therapeutics. Different regions on a given mPKS domain determine how it interacts both with its substrate and with other domains. Within the assembly line, these interactions are crucial to the proper ordering of reactions and efficient polyketide construction. Achieving control over these domain functions, through precision engineering at key regions, would greatly expand our catalogue of accessible polyketide products. Canonical mPKS domains, given that they are among the most well-characterized, are excellent candidates for such fine-tuning. The current minireview summarizes recent advances in the mechanistic understanding and subsequent precision engineering of canonical mPKS domains, focusing largely on developments in the past year.

## 1. Introduction

**Figure 1:**
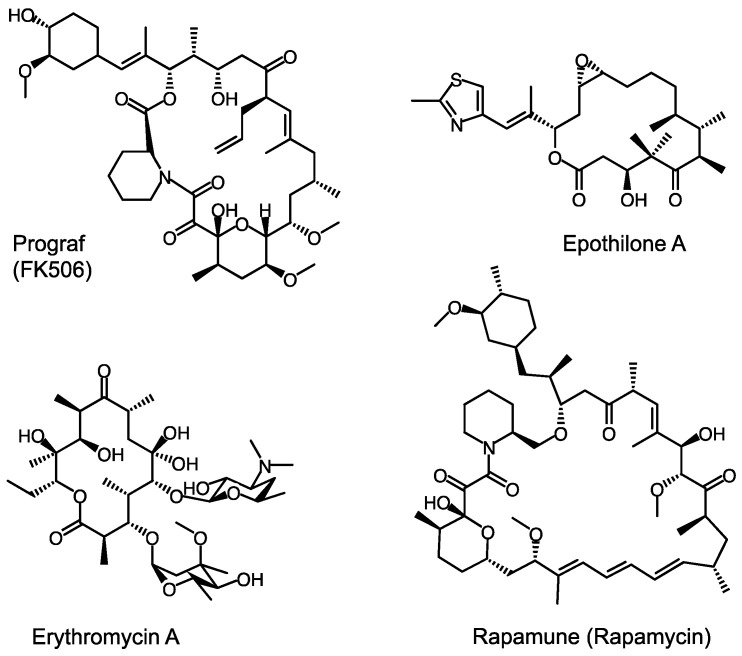
Bioactive polyketides with diverse structure produced by mPKSs [6,7].

Antibiotic resistance is on the rise, and it is essential to maintain a steady pipeline of antimicrobial candidates [1]. Microbial secondary metabolites are typically complex, chemically diverse and potently bioactive, and their biosynthetic pathways provide an industrially exploited resource for the discovery and manufacture of novel antibiotics [2,3]. Polyketide biosynthetic pathways are particularly promising, and have contributed numerous antibiotics currently on the market (Figure 1) [4]. Significantly, these pathways can be rationally engineered. The correspondence between the assembly line-like structures of modular polyketide synthases (mPKSs) and their polyketide products (referred to as their “collinearity”) has facilitated alteration of these megasynthases’ chemistries with high predictability [5]. The ability to alter polyketide products is particularly impactful in the context of therapeutics development: hits yielded by high-throughput screening often require modification and optimization before they can become drug candidates [6]. Consequently, the field of PKS engineering has matured dramatically over past two decades. While detailed and focused enzymatic studies are patently beneficial, the goal of efficiently engineering these megasynthases has also benefitted from keeping in-step with other developments in the field of PKS research—gaining from it new parts and better tools. This minireview gives a brief orientation to the overall field before diving into the most recent advances in fine-tuning control over canonical mPKS domains and modules.

## 2. A Whirlwind Tour of PKS Research

mPKS domain engineering has become one focus among many in the rapidly expanding field of PKS research [8]. Related “assembly line” families include non-ribosomal peptidases (NRPSs) and *trans*-AT PKSs. Iterative PKSs represent a related but non-assembly-line PKS class [9,10,11], very similar to animal fatty acid synthase (FAS). Lines between these families can be blurred: assembly line modules may also exhibit iterative behavior, and hybrid assembly lines can incorporate various components from different members of these related families [12,13]. Besides the canonical domains which produce basic molecular backbones for PKS and NRPS products, these families bristle with novel chemistries. Domains mediating unusual cyclizations, chain fusion and tailoring chemistries present new possibilities for generating chemical diversity [14,15], and bioinformatic algorithms have been developed to recognize some of these domains and tailoring enzymes within secondary metabolite clusters [11,16,17,18]. Recently-developed retrosynthetic algorithms now enable targeted genome mining based on desired product structure [19]. The evolutionary origins of PKSs and related families have been analyzed to gain insights regarding component compatibilities between different PKS pathways, as well as the natural combinatorial mechanisms that created them [20,21].

New assays, combined with modeling and structural visualization are enabling an increasingly detailed picture of mPKS function [22,23,24,25]. Moreover, similarity to other PKS and assembly line families permit further insights to be inferred for mPKSs as well [26,27]. mPKS engineering strategies themselves have evolved over the past decades, initially relying heavily on domain swaps, but now relying more on targeted, semi-rational and high-throughput approaches [2,6,28,29,30]. Finally, visions for high-throughput synthetic biology platforms have been described, using computational resources and automated microbiology to screen large quantities of yet-unexpressed natural products [31]. Achievement of such platforms could usher in a golden age of synthetic biology. Despite these advances, mPKS engineering still inadvertently results in productivity-draining disruptions that, from a biomanufacturing perspective, are calamitous [32,33,34]. Typically, no more than three modifications can be made to a PKS before polyketide production is reduced to trace amounts, and the unique challenges facing mPKS engineering are best understood by reviewing their architecture and function [12,24,35,36,37,38,39].

## 3. Polyketide Synthases: The Nuts and Bolts

**Figure 2:**
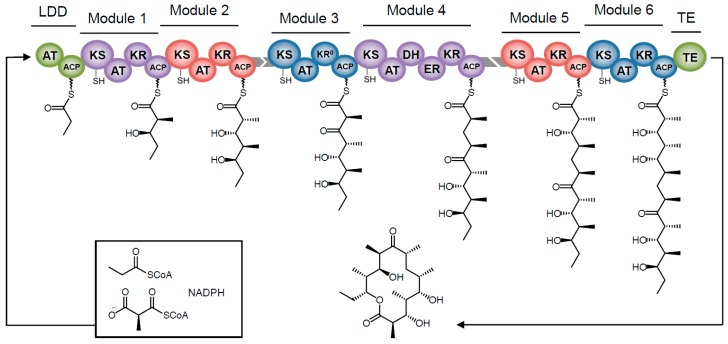
An example of mPKS architecture. This is a simplified diagram of the domain organization in of the 6-deoxyerythronolide B synthase (DEBS). Each module is colored differently, with domains indicated by labels, and docking domains colored in grey. Propionyl-CoA acts as a starter unit, and methylmalonyl-CoA is the elongation unit, added each time after chain is transferred from one module to the next. The thioesterase (TE) domain cleaves the acyl chain from the mPKS, sometimes causing macrocyclization. The KR° domain of DEBS M3 has lost its reductive activity, resulting in an unreduced ketone in the final product.

Type 1 mPKSs build functionalized carbon chains that can further react to form macrocycles and heterocycles. These megasynthases are composed of many catalytically active domains arranged in modular clusters (modules), with each independently-folding domain housing a catalytic center for a discrete enzymatic reaction (Figure 2). Modules typically contain three core ‘chain extension’ domains, which add keto-functionalized two-carbon units to an acyl chain intermediate. An acyltransferase (AT) domain is responsible for loading a chain elongation unit (typically malonyl- or methylmalonyl-CoA, though others are also possible). An acyl carrier protein domain (ACP) attached to a flexible ‘arm’ region shuttles the acyl chain to all other catalytic domains. The ketosynthase (KS) domain receives the acyl chain from the ACP, and condenses it with an extender unit. PKSs also have optional ‘chain processing’ domains, including a ketoreductase domain (KR) that reduces the keto group to a hydroxyl group, a dehydratase domain (DH) that eliminates the hydroxy group and a vicinyl proton to form a double bond, and an enoyl reductase domain (ER) that generates a completely saturated bond.

While some type 1 mPKS modules have been found to ‘stutter’—catalyzing two or three rounds of chain elongation [5,40,41,42]—in the most straightforward cases each module elongates the chain only once. The modules are arranged in a head-to-tail fashion, connected only by unstructured peptide linkers or discrete docking domains, creating the so-called ‘assembly line’. Following each round of chain extension, the ACP transfers the acyl chain intermediate to the KS domain of the downstream module. At the end of this modular assembly line, the chain is released and sometimes cyclized by a terminal thioesterase (TE). Since each module has a fixed position in the assembly line and contributes only once to the chain, each 2-carbon unit of the completed chain can be mapped to the module responsible for its incorporation. Accordingly, each module carries domains necessary to achieve the appropriate branching and reduction state for its particular chain unit. This enzyme-product collinearity, along with moderate tolerance for chain variation, has enabled targeted modifications to polyketide products and generation of combinatorial polyketide libraries [43].

## 4. The PKS Structure-Function Map: Then and Now

**Figure 3:**
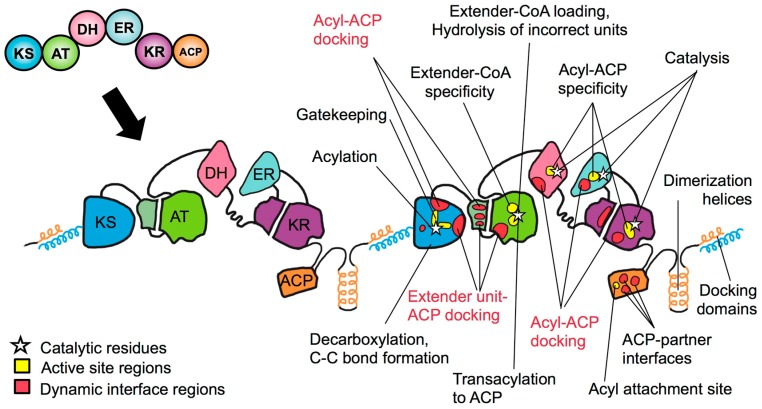
Transition to a more nuanced structure-function map of a polyketide synthase module. Earlier PKS engineering often presumed a functional modularity which mapped onto the architecturally modular domains of mPKSs. After many failed experiments, it became evident that a more nuanced understanding is necessary, if mPKSs are to be engineered predictably and efficiently.

While this description provides a generalized map of PKS architecture and function, it is admittedly oversimplified. It nonetheless formed the basis for the first generation of mPKS engineering, which envisioned mPKSs as having potentially ‘lego-like’ modularity [44]. Such an enzyme complex would have implied a one-to-one correspondence between domain and chemical trait in the polyketide product, and permitted facile interchange of modules and domains to easily and predictably generate a great variety of polyketides. Evidence for greater complexity underlying apparent mPKS modularity came in the form of many engineering failures, largely resulting from the use of the model described above: Skipped domains, stuttering modules, sub-optimal substrate specificity and disrupted protein-protein interfaces all contributed to significant losses in the activity and enzyme-product collinearity of engineered mPKSs [13,45]. These initial failures motivated more detailed investigations into mPKS structure and mechanisms, and the resulting insights are developing into to a more complex and predictive map of mPKS structure and function [46] (Figure 3).

It is now clearer that the contributions of the various domains to their canonical chemical outcomes are one of the many functions—chemical, structural and logistical—that they play. For example, a DH domain may be responsible for catalyzing a dehydration reaction within a given module, but it can only work on stoichiometric amounts of substrates that have been reduced to the correct stereochemistry by an appropriate KR domain [47], and perhaps only after it interfaces correctly with its cognate ACP [48]. Therefore, the chemical outcome of a double bond on a polyketide product depends not only on the presence of a DH in the correct module, but also the presence of a suitable KR and an appropriate ACP/DH interface. Similarly, the nature of the ACP/KS interfaces within a module can influence a its capacity for iterative (or stuttering) behavior [42]. Such behavior may be held in check by the ‘gatekeeper’ function of one of the KS binding pockets [49], which sometimes discriminates between acyl chains of different size and stereochemistry. Chain length and stereochemistry can further affect the catalytic rate and activity of other mPKS domains.

Overall, multiple chemical, structural and logistical functions influencing a single chemical outcome are performed by different regions within various architecturally discrete domains. From an engineering standpoint, it is critically important to decipher which of these functions play the most predominant roles for a given chemical outcome, and assess the degree to which they can be tuned independently from their parent domain’s other functions. To this end, several engineering approaches are being honed to alter one function while minimizing disruption to other others within same domain and module, including active site ‘surgeries’ and more refined domain swaps. Key lessons from recent work will be underlined as ‘take-home messages’ for the prospective mPKS engineer.

## 5. Snapshots and Dynamics of Concerted Module Mechanisms

**Figure 4:**
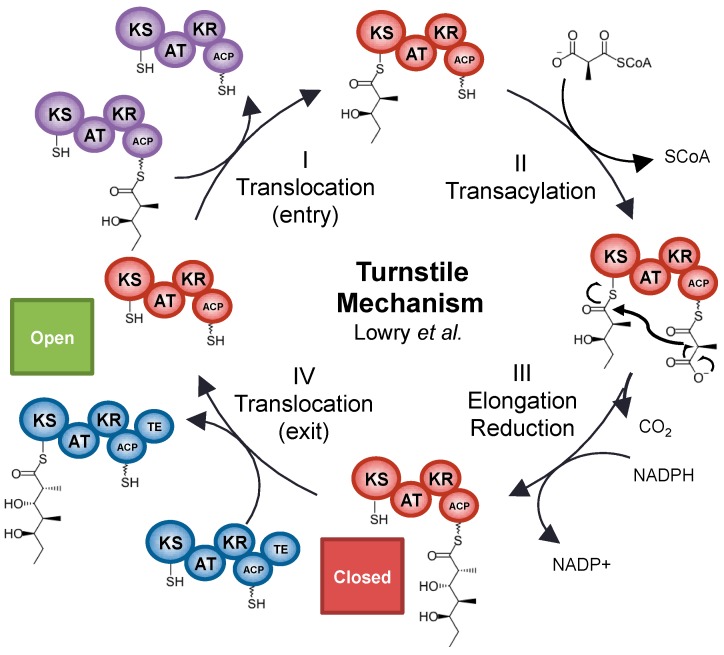
Operation of the turnstile mechanism during a catalytic cycle of an mPKS module. Step I: The KS of a module receives a diketide from the upstream module’s ACP. Step II: a methylmalonyl extender unit is attached onto the current module’s ACP. Step III: the ACP brings the elongation unit to the KS, which catalyzes elongation. The ACP then removes the new triketide, and shuttles it to other reducing domains. Although the KS is empty, it cannot accept another chain until Step IV: when the ACP of the current module translocates the acyl chain to the next KS.

Recent cryo-EM, SAXS, and crystal structure-based visualizations of whole-module and multimodular PKS architectures [36,37,50,51,52,53] have enabled exploration of mechanisms that operate at the level of the whole module. The dramatic conformational changes revealed in the cryo-EM images of the PikAIII module published by Dutta et al. and Whicher et al. provide strong evidence that modules undergo dynamic, concerted actions to mediate appropriate substrate processing [37,50]. Just prior to the publication of these cryo-EM based architectures, Khosla et al. published their hypothesis about a turnstile mechanism for governing chain processing in assembly line synthases [54] (Figure 4), an idea consistent with the concerted module movements observed in the cryo-EM structures. Both the cryo-EM maps and the turnstile hypothesis have significant implications for polyketide synthase engineering, since it is now clear that linkers and domain surfaces cannot be assumed to play simply structural roles, but instead they may mediate large conformational changes or relay conformational information to other domains.

More evidence has been recently been published to support the hypothesis of a turnstile mechanism, namely, the single substrate occupancy of PKS modules [55]. Using a series of radiolabelling assays, it was demonstrated that modules possessing an acyl substrate on their ACP are incapable of loading an ACP-borne diketide substrate onto their KS. Only when the substrate is released from the ACP could the KS receive another acyl chain. It is still unclear how ACP loading state information is transferred to the KS, but the conformational changes observed in the cryo-EM models suggest that information transfer could be initiated with the acylation of the KS. Such nuanced functions may call for more careful engineering, with special attention to the yet-known roles potentially played by interdomain linkers in mediating such “state” information [37].

It should be noted, however, that there remain ambiguities in the cryo-EM structures—while they were prepared to mimic various reaction steps in an elongation cycle, the conditions weren’t replicated with complete correspondence—particularly, extra or absent acyl substrates could have plausibly had unnatural impacts on module conformation [24]. Moreover, since an earlier FAS-like model is also well-supported, it remains unclear which should be taken as the most representative of model of module architecture [24,36]. This is especially significant for understanding KS-ACP interfaces, which have only been well-mapped from the from the ACP side [56,57,58]. Therefore, it is instructive to consider engineering approaches with respect to both the cryo-EM and FAS-like models.

## 6. Ketosynthase: Key Players in Rate and Promiscuity

**Figure 5:**
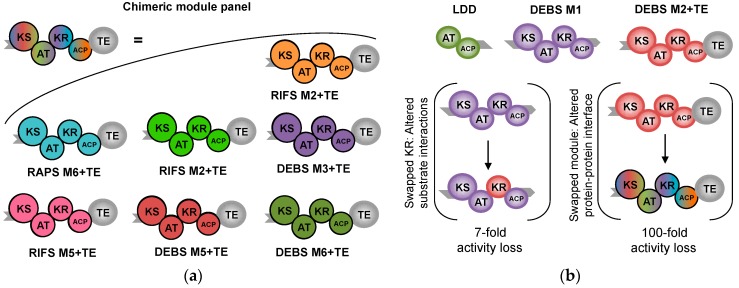
Layout of the chimera experiment by Klaus et al. (**a**) denotes a panel of modules to interact with DEBS module 1; (**b**) denotes the experimental setup: A thioesterase was added to the second module of DEBS, creating a mini-PKS producing a truncated product. First, module 2 (M2) was swapped with nine other modules, all natively processing substrates with the same B1 stereochemistry near the thioester. Since this manipulation controlled stereoselectivity, they reasoned that the main variation would be caused by protein-protein interactions. Next, the same set of experiments were repeated, this time with the KR from M2 carefully swapped into that of M1, resulting in products of altered stereochemistry. Their results suggested that non-natural protein-protein interactions were a much greater threat to productivity than the protein-substrate interactions.

Given the strong role of ACP-KS interactions in this turnstile mechanism, as well as in the activity outcomes of module-swapped PKS chimeras [33,59,60], the contribution of KS/ACP interactions to overall turnover rate is a question of considerable interest. To investigate this, Klaus et al. designed a set of experiments to elucidate the relative contributions of protein-protein interactions and protein-substrate interactions on turnover rates in chimeric mPKSs (Figure 5). A panel of PKS modules processing acyl substrates with the same α,β-stereochemistry but originating from different PKSs was assayed to assess their ability to accept a diketide substrate from the same donor module [61]. The difference in turnover rates, which mainly reflect the extent of compatibility at the chimeric ACP/KS interfaces, ranged from 10–100-fold. In contrast, the same experiment performed with modification to the diketide substrate resulted in just a 10-fold change in activity. Overall it suggests that a protein engineer should prioritize protein-protein interactions, consistent with the AT_n_-KS_n+1_ approach for combining modules [59].

One intriguing behavior observed in the cryo-EM models was the role of the acyl chain in influencing the interaction between the upstream ACP and its KS partner. Without an acyl chain, the ACP would not even contact the KS, while its presence enabled sufficient affinity. Questions raised about the role of the substrate in ACP-KS interactions—and ACP-partner interactions, more generally—are being further investigated. Recently, NMR studies tracking the interaction of ACPs with their acyl substrates have shown that larger chains interact extensively with their carrier protein, possibly nestling within a hydrophobic pocket while leaving the thioester component displayed. This could position the thioester β-carbon for easy access and recognition by partner domains, enabling their reduction state to tip ACP affinity towards the appropriate partner [62].

**Figure 6:**
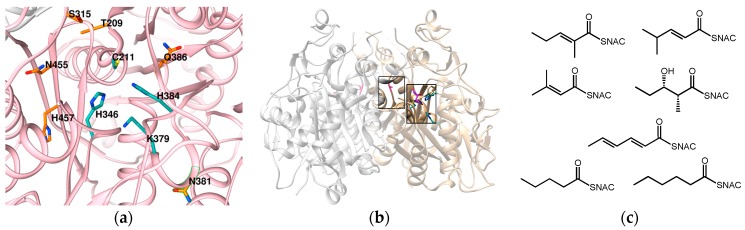
(**a**) Homology model of the active site of KS1, prepared using SWISS-MODEL [64] with DEBS KS3 as a template. Catalytic residues are in cyan, and the orange residues are conserved residues that were also investigated by Robbins et al.; (**b**) Location of alanine residue on the DEBS KS3 dimer interface loop (PDB: 2HQO3). Mutation of this alanine to a tryptophan greatly expanded the substrate range of DEBS KS3; (**c**) Seven new substrates that previously could not be processed by DEBS KS 3. Note that the alanine (pink) is farther away from the active site (shown with residues colored cyan and purple).

While KSs of canonical (*cis*-AT) mPKSs are known to have relaxed substrate specificity [38], they can be stringent towards certain features, particularly stereochemistry [63]. Perhaps in light of the ACP/KS interface studies described above, another recent study focused on mapping the function of conserved residues in the DEBS KS1 active site. *Cis*-AT mPKS KS domains have been relatively underexplored with respect to engineering and active site residue characterization, possibly because of its involvement in so many different functions. To narrow down the selection of active site residues to explore, Robbins et al. compared 90% consensus sequences from 226 non-iterative PKSs with that of 24 iterative ones [49]. Conserved residues between one or both groups within a 10 Å of the active cysteine were selected for further analysis (Figure 6). Results from standard SDM and innovative assays have implicated a pair of histidine residues in mediating ACP interactions, as well as a lysine residue in controlling C-C bond formation. An asparagine residue was also identified as possibly playing a role in the turnstile mechanism, since its mutation results in PKS modules carrying more than one acyl chain simultaneously.

Even as this active site exploration was underway, Murphy et al. were also exploring KS active site residues, seeking to identify promiscuity-conferring active site mutations for DEBS KS3 (Figure 5). Candidate residues in DEBS KS3 were chosen through comparison to the KS active site residues of mycolactone synthase (MLS) modules [65] which share >97% sequence identity, despite processing a substrates with a variety of stereochemistries and functionalization. Eight residues were swapped, and while some displayed modest improvements in activity and substrate promiscuity, one swap—an alanine to tryptophan swap in the active-site proximal dimerization loop [66]—resulted in dramatically increased activity and the ability to process eight new substrates. Taken together, these recent advances constitute pioneering work in mapping out various KS functions and their relative contributions to chemical outcomes. The degree to which these functions—ACP compatibilities, substrate selectivity and chain elongation—can be tuned independently remains to be determined.

## 7. Acyltransferase: Specificity over Substrate Promiscuity

Native promiscuity, inactivation and complementation, domain-swapping and active-site surgery have all been employed to exploit acyltransferases as decision gates for extender unit incorporation [6]. These approaches have enabled the incorporation of many non-native extender units into polyketides, and are reviewed in greater detail elsewhere [2,6,67]. Such approaches, while succeeding in non-native unit incorporation, often do so with reduced activity: domain-swapping disturbs interdomain linkers or interfaces, and SDM-induced promiscuity has mainly been achieved by reducing affinity for a native substrate [68]. Therefore, it remains a challenge to alter substrate specificity without damaging activity.

**Figure 7:**
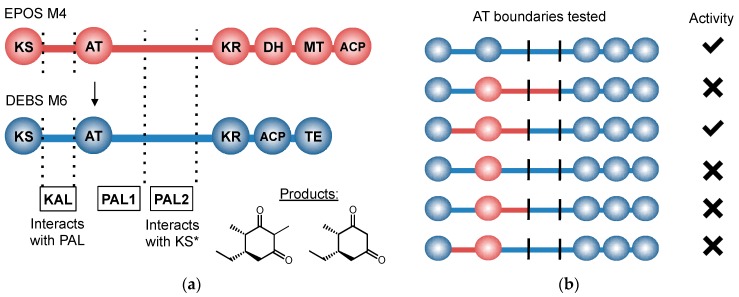
(**a**) Boundaries selected for AT-swapping experiments by Yuzawa et al. and the (**b**) outcome of assorted boundary combinations. Activities reported are relative to that of wild-type. KAL and PAL denote KS-AT linker and post-AT linker, respectively.

Yuzawa et al. recently re-evaluated fusion points for AT domain swaps enable incorporation of AT domains with unusual substrate specificity (Figure 7) [69]. Since domain swaps in previous investigations did not rigorously explore the role of interdomain linkers, they selected a set of fusion points along the KS-AT (KAL) and post-AT (PAL) linkers of DEBS M6, which would act as a receiver for an AT domain from epothilone synthase (EPOS). Fusion point selection was informed by sequence conservation, structural insights and key interactive residue locations along the linkers [70]. The most promising fusion point combinations yielded nearly 70% wild-type activity, and included the KAL, as well as the less well-conserved portion of the PAL. This was rationalized with reference to AT-KS crystal structures, in which part of the post-AT linker rejoins the KS before continuing on the KR. This approach was then expanded to a unimodular system based on lipomycin synthase, in which several alternative ATs were incorporated with wild-type activity retained.

Theoretical investigations of specificity determinants in DEBS AT6 have also finally enabled an alteration in AT preference from its natural substrate an unnatural one—propargylmalonyl-CoA [71]. Previous application of SDM towards AT domains often achieved promiscuity through reducing affinity for natural substrates, or—as recently done with avermectin—simply by removing steric obstruction [10,72]. In contrast, this novel inversion of specificity was achieved through the preparation of saturation mutagenesis at specified residues within the active site of AT domain, and screening using a novel in vitro competitive elongation assay. In this assay, two extender units compete for loading and extension with a provided diketide-SNAC. Preference for this unnatural unit was enhanced by sevenfold, leading it to produce wild-type proportions of a propargylated macrolide, 88% of the total product in competitive assays.

These recent investigations show a redirection of focus away from enhancing substrate promiscuity and towards controlled tuning of AT specificity. Ideally, AT residues involved in ACP interactions could also be tuned for enhanced compatibility, since older research has suggested up to a 10-fold loss in activity caused by a mismatched AT-ACP pair [72]. Overall, the approach employing saturation mutagenesis and competitive assays seems to hold more potential for introducing modifications with minimal activity loss, but constitutes a more labor-intensive approach than the former improved domain swap method.

## 8. Ketoreductase: Modular Context and Catalysis

**Scheme 1:**
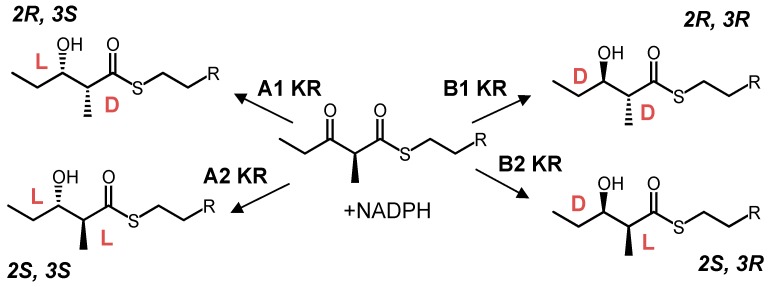
Four stereochemical configurations are derived from four types of ketoreductase (KR). A2 and B2-type KRs epimerize their substrates in addition to reducing them.

While ketoreductases are known to have broad substrate tolerance [73,74], the degree to which they act on various substrates, as well as the resulting product stereochemistry, is neither constant nor discrete [74,75,76]. Stereochemical control in polyketide assembly lines has also recently been reviewed more in-depth [7,76]. KRs appear to behave most reliably towards their native substrates and within their native context, but nonetheless remain a target for PKS engineering, especially for their potential to control stereochemistry. Active-site surgery and reducing loop domain swaps (RLS) both present viable options for ketoreductase engineering [6,77,78]. As many KRs appear to be robust towards chain length and branching, a key aim in KR engineering is to control the stereochemical outcome of the reduction—both for the resulting hydroxyl group and also for any branch at the α-carbon. KRs can be categorized into 6 types—A1, A2, B1, B2, C1 and C2. A and B denote the stereochemistry of the β-alcohol group, namely levo (l) and dextro (d) respectively. A1-KRs accept substrates bearing a d-α-branch, though the A2-type may accept an α-branch in either configuration but will epimerize it to the L isomer. Type-B KRs can be categorized analogously (Scheme 1). C1 represents an entirely inactive KR, while C2 KRs cause epimerization without ketoreduction [79]. Signature motifs can be used to predict KR stereochemistry, however, modification of a KR’s stereochemistry type based on these motifs has only been successful in some isolated KR fragments, and not for all stereochemistry types [80].

The RLS method, on the other hand, introduces KRs or sets of reducing domains from a single donor into the host module sequence at conserved locations, and has provided an efficacious way to alter some KR stereochemistries [78]. Earlier studies showcased the method’s ability to carry out swaps between non-epimerizing KRs, however, it has been less successful in importing epimerizing ones. A recent RLS study focused on exchanging non-epimerizing A1-KRs with a panel of epimerizing A2 and B2 KRs confirmed the limitations of this method to introduce functional epimerase activity. Out of the sixteen epimerizing KRs swapped into the DEBS KR6 position, only two retained any activity [81]. A current hypothesis based on proteolytic analysis of modules with epimerizing and non-epimerizing KRs suggests that epimerizing modules may possess a more open architecture, which is important for proper KR functioning.

The reductive catalytic machinery of KR domains has also been mapped [79], and recent work has confirmed that the tyrosine and serine residues responsible for epimerization are the same as those used ketoreduction, although a general base for proton abstraction has not yet been identified [82]. This suggests that non-catalytic residues play a significant role in determining stereochemical outcomes. A KR products’ stereochemical outcome is ultimately determined by the orientation of the acyl chain with respect to its NADPH cofactor, which is held in the same position across all reducing KR types. While some high-throughput and rational SDM have successfully altered stereochemical outcomes [75,83], the limitedness of such instances show that further yet unexplored determinants are involved.

**Figure 8:**
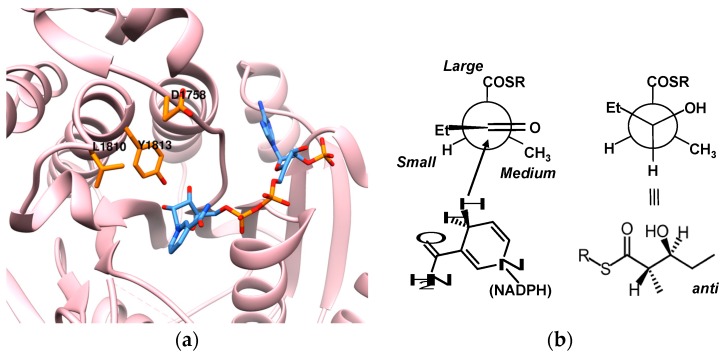
(**a**) B2-activity-conferring residues for 6-DEBS KR1 (PDB: code 2FR1). Replacement of Leu and Asp residues with alanine resulted in increased turnover rates yielding products with A2 stereochemistry. Residues in orange, NADPH is in blue; (**b**) In non-A2 KRs, the proportion of A2 products is often elevated following disruption of certain active-site residues, regardless of original KR chemistry. This trend can be rationalized by Felkin-Anh transition states, which show that A2 stereochemistry results from the least sterically hindered transition states.

A recent study sought to determine whether substrate positioning was achieved primarily through interactions between the KR and the acyl chain itself, or whether the phosphopantetheine (Ppant) arm holding the acyl chain was implicated [84]. Beyond revealing that the Ppant arm did not heavily mediate orientation, the study also discovered that for all stereochemical KR types, active-site expanding mutations caused an increase in proportion and production rate of type-A2 products (Figure 8). Out of a panel with all four KR types, the alteration of B2 to A2 stereochemistry was particularly pronounced. This has led to the hypothesis that A2 stereochemistry results from the lowest-energy state for ketoreduction, and will be the favored product in the absence of positional constraints. The implication for PKS engineering is that it should usually be easier to convert other stereochemistries to A2 simply by removing position-constraining active site residues.

This hypothesis has been supported by the results of another study, one focused on improving outcomes for the RLS approach. Eng et al. noticed that a functional sequence discrete from the KR domain (termed dimerization element, DE [85]) was usually included in the donor loop swapped into the host module. While initially described as a small ordered sequence meant to stabilize interactions between a module’s dimers, this role would be incongruous with the cryo-EM account of polyketide synthase architecture. Nonetheless, it was postulated that conserving the host module’s dimerization element could enable and improve activity. In both instances, this modification resulted in 3-fold increases in activity as well as the formation of more structurally sound modules [86]. Omission of the DE resulted in insoluble modules. Using this method, Eng et al. swapped a panel of A1-KR donors with the A2-KR position of lipomycin PKS M1, and assayed them with small-chain substrates. While all KRs whose native substrates were small chains faithfully conferred the less-energetically favored A1 stereochemistry, 6-DEBS KR6, optimized for a longer chain, produced both A1 and A2 products. Presuming a larger active site to fit a larger chain, this outcome agrees with the earlier hypothesis of decreased constraints on the acyl substrate favoring an A2 stereochemical outcome.

These advances in re-engineering the stereochemical outcomes of the KR domain has been complemented by investigations into its ACP specificity. The interaction landscape of four reducing KRs from DEBS and five ACPs from DEBS was assessed in a simple in vitro assay [87]. The outcomes showed that KRs often exhibit a modest preference for their cognate ACPs by a factor of 10- to 50-fold, although this rule was violated by KR6, which preferred ACP5 instead. Therefore, an ACP/KR mismatch, particularly between components belonging to the same PKS, should not be individually responsible for complete inactivation. Overall, recent work in understanding and engineering ketoreductase functions has achieved considerable progress and control. While it seems that controlling B2 and A2 activity via RLS will require a more detailed understanding of module host prerequisites, now A2 to A1 swaps have been achieved through a modified RLS approach [86], and active site surgery has enabled conversion of B2 to A2 stereochemistry [84].

## 9. Dehydratase and Enoylreductase: Minimally Controlled

There has been comparatively less progress recently in honing control over dehydratases. Most research on these domains currently proceeds at the level of structure, substrate specificity, protein-protein interactions and reaction outcomes [7,79]. The most relevant engineering techniques applied to them have been their incorporation into RLS. The dehydration reaction can be achieved through syn- or anti-elimination, and can generate either a *trans* or *cis* product (the latter typically more rare). *Trans* double bonds typically result from B-type ketoreduction, while *cis* double bonds are sometimes formed from A-type products [47]. While sometimes robust to varying chain lengths, DHs tend to be diastereospecific, often accepting only one out of four possible diastereomers [48]. Reversal of stereochemistry in substrate tolerance has been observed when comparing SNAC substrates or non-native ACP-bound substrates, thereby revealing a dependence on interactions with its cognate ACP [88]. Dehydratase swaps can be done successfully, however, as demonstrated in part of a larger effort to develop an adipic acid-producing polyketide synthase [89].

There have been few recent developments in mPKS ER engineering. Besides a well-known Tyr-to-Val mutation, which alters methyl branch orientation from (2*S*) to (2*R*) [90], there has also been progress in trapping FAS ACP-enoyl reductase interfaces kinetically and crystallographically [91]. Because of their similarity to mPKS domains, useful insights could possibly be inferred from such structural information.

## 10. Thioesterases: Determinants of Macrocylization

Thioesterase sequences vary as much by phylum as they do by PKS family type, and factors determining their two typical choices for substrate offloading, hydrolysis or macrocyclization, remain unpredictable by sequence alone [92]. Although they do not appear to interface significantly with the acyl-bearing ACP, many thioesterases have evolved sensitivity towards specific features on a polyketide chain [24,93]. These can ultimately determine offloading outcome. One outstanding question in thioesterase engineering concerns the nature of the binding site features that underlie the TE decision to hydrolyze or macrocyclize. The role of a lid loop, which exists in several thioesterases, is also a mystery. Finally, it is not yet clear whether some combination of sequence and structure could be used to predict macrocyling TEs from TEs that simply catalyze hydrolysis. Efforts to co-crystallize a TE with macrocycle-like inhibitors are ongoing and will undoubtedly reveal valuable insights once successful [94]. TEs have also been assayed with various substrates as an empirical way of elucidating their recognition features. One recent study identified a non-native substrate that forced the pikromycin TE to become ‘indecisive’—producing both linear and cyclized products [95]. This could be an interesting system for further study, as there are no accounts of naturally ‘indecisive’ thioesterases.

In the absence of clear crystal structures, detailed theoretical approaches have been undertaken to model both cyclization and hydrolysis reactions catalyzed by DEBS TE. Chen et al. used QM and MM models to investigate determinants of the reaction fate of three DEBS-like SNAC substrates [96]. Their work suggests that the reaction is mediated through an induced-fit mutual recognition between the TE enzyme and substrates. A high-energy ‘pre-reaction’ state is achieved by the substrate destined for macrocyclization, which is mediated and stabilized by hydrophobic interactions and an H-bond network in the loop region. Substrates lacking the key recognition features were unable to form those critical H-bond and hydrophobic interactions, which precluded macrocyclization. The resolution at which this reaction was modeled thus speaks to all three of the field’s questions, providing useful hypotheses for further research.

## 11. Control beyond Single Domains: Extensive, Methodical mPKS Module Engineering

A n unusually methodical domain swapping approach has been demonstrated in recent work by Hagen et al., in their development of an adipic acid-producing monomodular PKS [89]. This chimeric PKS was developed from the first module of borrelidin synthase (BORS), which is distinct in its ability to utilize succinyl-CoA as a starter unit. To produce the platform chemical adipic acid, it required only an additional TE and ER domain.

**Figure 9:**
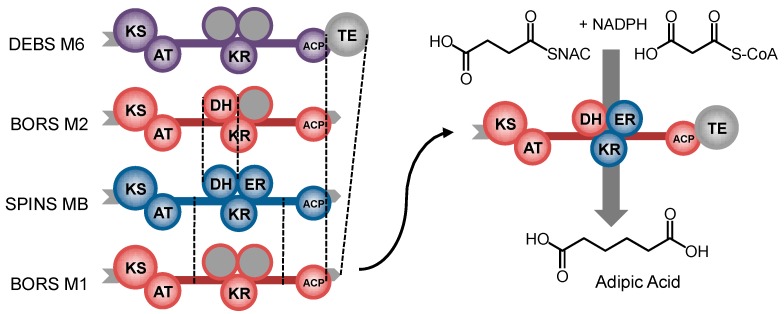
Procedure undertaken by Hagen et al. to develop an adipic acid-producing PKS module. Dashed lines denote the multiple junctions tested for each loop and domain swap.

Since the RLS method has been the most successful in exchanging or adding reducing domains, reducing loops from four alternative modules were tried using two different junctions, giving a total of 8 chimeric modules. These chimeras were evaluated in vitro for their ability to elongate succinyl-CoA with a malonyl extender unit. Using a mass-spectrometry-based phosphopantetheine ejection assay [97], which enables detection of stalled intermediates, they found that two of the chimeras were incapable of elongating the succinyl starter unit, and the remaining six chimeras stalled at the dehydration step. Reasoning that the succinyl carboxyl group was inhibiting the non-native DHs, an alternative DH from the second BORS module that dehydrates a substrate analogous to succinyl-CoA was selected for incorporation. They first confirmed this hypothesis by demonstrating that BORS M2 could dehydrate the 3-hydroxyadipoyl-ACP intermediate by assaying its activity as a standalone domain. Then three junctions were evaluated for incorporation of BORS DH2 into the BORS M1 chimeras, and one of them, for a chimera with spinosyn reducing loop domains, was found to be effective (Figure 9).

The TE domain was added following the aforementioned troubleshooting and refinement, and the following in vitro assays revealed the final chimera to produce adipic acid at concentrations comparable to the amount of 3-hydroxyl-adipic acid produced by wild-type BORS M1-TE. This work demonstrates a remarkably methodical approach to PKS engineering through domain swaps, testing panels of parts, evaluating multiple junctions for each exchange, and evaluating performance of each domain through detection of reaction intermediates. We anticipate widespread use of this method in the years ahead, although its in vitro nature likely precludes high-throughput application in the short term.

## 12. Conclusions

Significant progress has been made in mapping and fine-tuning the functions of various canonical PKS domains. The presence of a turnstile mechanism shows that the fusion points of any domain swaps should be chosen judiciously, and several options should be attempted. The KS plays a key role by interfacing with the translocating ACP to receive its acyl substrate. If the interfaces are incompatible, the loss in activity is quite significant, while incremental modifications to the substrate had a smaller effect on productivity. Within the KS, catalytic and supporting residues have been mapped and we now know that a mutation in the active-site proximal dimerization loop greatly enhances activity and substrate tolerance. ATs have been extensively engineered to enable incorporation of non-native building blocks, but often through inefficient means: domain swaps have typically caused disrupted protein interfaces, and SDM methods achieved promiscuity only by destroying natural substrate affinity. However, recent work honing both these methods have resulted in mutants and chimeras with swapped instead of damaged activity. Key determinants like modular context and binding site size have been shown to influence KR outcomes, and an overlooked role of the dimerization element in reducing loop swap outcomes has been demonstrated. While the remaining domains have seen more characterization and less engineering, TE theoretical studies have developed insightful models that provide hypothesis for further verification. Finally, an engineering tour-de-force has demonstrated how mPKS modification can be undertaken in a stepwise, methodical fashion to produce high-activity chimeras with intended catalytic ability. These advances illustrate valuable engineering principles for modular polyketide synthase engineering, and will likely enable more extensive, predictable, and independently tunable modifications.
